# Imagined motor action and eye movements in schizophrenia

**DOI:** 10.3389/fpsyg.2013.00426

**Published:** 2013-07-12

**Authors:** Céline Delerue, Muriel Boucart

**Affiliations:** Laboratoire de Neurosciences Fonctionnelles et Pathologies, Centre National de la Recherche Scientifique, Hôpital Roger Salengro, Université Lille – Nord de FranceLille, France

**Keywords:** schizophrenia, eye movements, visual exploration, imagined motor action, attention

## Abstract

Visual exploration and planning of actions are reported to be abnormal in schizophrenia. Most of the studies monitoring eye movements in patients with schizophrenia have been performed under free-viewing condition. The present study was designed to assess whether mentally performing an action modulates the visuomotor behavior in patients with schizophrenia and in healthy controls. Visual scan paths were monitored in eighteen patients with schizophrenia and in eighteen healthy controls. Participants performed two tasks in which they were asked either to (1) look at a scene on a computer screen (free viewing), or (2) picture themselves making a sandwich in front of a computer screen (active viewing). The scenes contained both task-relevant and task-irrelevant objects. Temporal and spatial characteristics of scan paths were compared for each group and each task. The results indicate that patients with schizophrenia exhibited longer fixation durations, and fewer fixations, than healthy controls in the free viewing condition. The patients' visual exploration improved in the active viewing condition. However, patients looked less at task-relevant objects and looked more at distractors than controls in the active viewing condition in which they were asked to picture themselves making a sandwich in moving their eyes to task-relevant objects on an image. These results are consistent with the literature on deficits in motor imagery in patients with schizophrenia and it extends the impairment to visual exploration in an action imagery task.

## Introduction

Both behavioral (Ellis and Tucker, 2000; Tucker and Ellis, 2001, 2004) and brain imaging (Chao and Martin, 2000; Gerlach et al., 2002; Grèzes and Decety, 2002; Grèzes et al., 2003) studies have shown that objects, or photographs of objects, potentiate the appropriate action and that this process involves activation of motor representations and depends on the intention of the observer. Even in the absence of explicit intentions to act, attending to an object, or searching for an object, may activate motor representations appropriate to reaching, grasping, and manipulating the object (Tucker and Ellis, 1998; Bekkering and Neggers, 2002; Grèzes et al., 2003).

The aim of the present study was to investigate whether the mental imagery of an action sequence modulates the visuomotor behavior in patients with schizophrenia and in healthy control participants. Orientation of attention and eye movements have been shown to depend both on the characteristics of the stimulus (stimulus driven) and on the goals and intentions of the observer [goal driven; see Henderson (2003) for a review]. We assessed if the pattern of eye movements varies as a function of whether the observer mentally performs an action or not. Visual exploration of photographs of scenes was compared in two conditions: one in which participants were required to picture themselves interacting with several objects (active viewing) and one in which they were asked to just look at a scene (free viewing).

Motor imagery is a dynamic state in which an individual simulates mentally the performance of a specific motor action (Decety, 1996; Jeannerod, 1997). Behavioral studies in healthy participants show that real and imagined motor actions are subject to the same environmental and physiological constraints (Decety, 1996; Jeannerod, 1997; Maruff et al., 1999). Neuroimaging studies have reported common activation patterns in the mental simulation and the real motor actions (Decety et al., 1994, 1998). Moreover, motor imagery requires participants to generate an internal representation of intended but unexecuted motor actions and to anticipate the consequences of that action as if it had really been carried out (Jeannerod, 1997). Tasks of motor imagery may therefore provide one means by which the self-monitoring of goal directed actions can be examined in patients with schizophrenia.

Several studies have reported eye movement disturbances in schizophrenia, such as deficits in anti-saccade tasks (Harris et al., 2009), in smooth pursuit (Clementz and Sweeney, 1990; Sweeney et al., 1998), and in visual exploration. Visual scan path impairments have even been proposed to serve as a trait marker for schizophrenia (Loughland et al., 2002a, 2004). The majority of these studies have been conducted with faces as stimuli. Patients with schizophrenia usually make fewer fixations than healthy controls on salient facial features (eyes, nose, and mouth) and their exploration duration is reduced. Restricted visual scan path have also been reported with other, less socially relevant stimuli, like geometrical shapes (Kojima et al., 1992; Obayashi et al., 2003); Rorschach stimuli (Minassian et al., 2005), and photographs of landscapes, meaningless textures, and fractals (Bestelmeyer et al., 2006). In most of these studies, visual scanning has been examined under free viewing conditions (Green et al., 2003; Bestelmeyer et al., 2006) and for some of them (Streit et al., 1997; Loughland et al., 2002a,b), participants were asked to determine the facial expression.

Based on these findings, especially the reduced visual exploration, the fact that patients with schizophrenia exhibit significant difficulties with planning and organization of action (Pantelis et al., 1997; Jogems-Kosterman et al., 2001; Delevoye-Turrell et al., 2006, 2007), and that these patients show an exaggerated susceptibility to distraction (Ducato et al., 2008a,b), we expected patients to explore less than controls in the free viewing condition and to exhibit a larger amount of fixations to the task-irrelevant (distractor) objects in the active viewing condition. In this study, we also used motor imagery in patients with schizophrenia to assess whether the generation of an internal image of intended actions would be impaired in schizophrenia.

## Materials and methods

### Participants

Eighteen medicated in- and out-patients fulfilling the DSM-IV diagnostic criteria for schizophrenia (American Psychiatric Association, 1994) and 18 healthy control participants without a psychiatric diagnosis (axis I and II) and without a family history of mental illness took part in the experiment. Control participants did not take any medication at the time of test. Patients were recruited in the Department of General Psychiatry in Lille University Hospital, the Department of General Psychiatry at Arras General Hospital and the Psychology Center in Béthune (all located in northern France). Control participants were age and sex-matched students and members of the medical staffs of the different hospitals. Inclusion criteria for all groups were normal or corrected-to-normal vision (assessed by the Snellen chart). Exclusion criteria were recent history of substance abuse, ocular disease, epilepsy and other neurological disorders, and failure to understand the instructions. After interview, schizophrenic symptoms were rated using the Positive and Negative Syndrome Scale (PANSS; Kay et al., 1986). The study was approved by the local Ethics Committee. Informed consent was obtained from all participants. Group characteristics are summarized in Table 1.

**Table 1:** **Means and standard deviations (*SD*) of participant demographics**.

	**Patients *N* = 18**	**Controls *N* = 18**
	**Mean (*SD*)**	**Mean (*SD*)**
Age (years)	35.7 (7.0)	34.2 (8.7)
Gender (Male/Female)	15 M / 3 F	15 M / 3 F
Antipsychotic medication (mg chlorpromazine equivalent )	449.9 (203.3)	–
Benzodiazepine medication (mg diazepam equivalent )	12.7 (6.0)^*^	–
Illness duration (years)	11.1 (8.0)	–
PANSS positive symptom score	19.2	–
PANSS negative symptom score	21.5	–
PANSS general psychopathology score	37.7	–
Total PANSS score	78.4	–

* 12 out of 18 patients were treated with benzodiazepine medication.

### Stimuli

**Figure 1:**
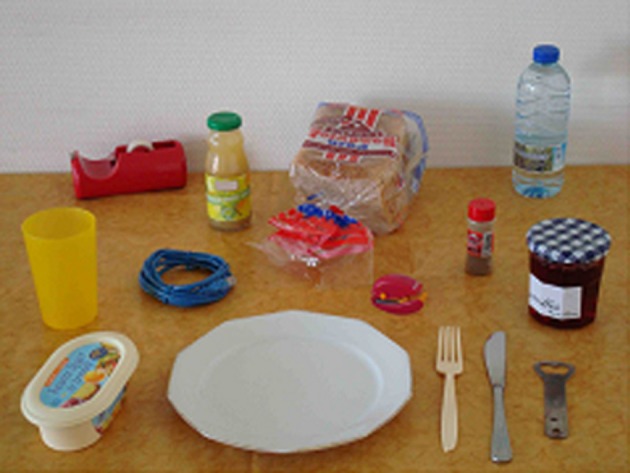
**Example of scene layout used in this experiment**. The scene contained both 7 task-relevant *(bread, butter, jelly, knife, plate, glass, water bottle)* and 7 irrelevant objects.

Different scene layouts were built containing task-relevant objects (*n* = 7) required to make a butter and jelly sandwich and to pour a glass of water, and task-irrelevant objects (*n* = 7). All objects were laid out on a table. When the participant was seated at the table, with all objects within reach, the plate close to the observer subtended about 20° of visual angle, and the butter and jelly subtended about 7°. All objects were located within a region covering 90°. These scenes were then photographed. The scene pictures were 768 pixels in height and 1024 pixels in width, and subtended a vertical visual angle of 21° and a horizontal visual angle of 29° at a viewing distance of 60 cm (Figure 1).

### Equipment

Visual scan paths were collected monocularly. Monocular (right) eye position was monitored using the iViewX™ HED from SensoMotoric Instruments (Teltow, Germany) eye tracker with a scene camera. The video based eye tracker was head-mounted, using infrared reflection to provide an eye-in-head signal at a sampling rate of 50 Hz and accuracy of 1°. The scene camera mounted on the head was positioned so that its field of view was centered on the subject's field of view. Calibration was performed using a five-point grid. Following calibration, the eye tracker creates a cursor, indicating eye-in-head position, which was superimpose on the scene video. This cursor was just for purposes of analyses but it was not visible for the participant. The scene camera moved with the head, so the eye-in-head signal indicated the gaze point. The eye tracker thus provided a video recording of eye position from the participant's perspective on the scene and the data analysis was based on the video, as there was no separate numerical data stream. The video recordings were analyzed on a frame-by-frame basis, recording the time of initiation and termination of each eye movement, and the spatial locations of the fixations. Saccades appeared in the large displacements of the cursor between video frames. The beginning and end of each saccade was identified and recorded using a video analysis tool. Fixations were defined visually when the cursor stayed within a given location (less than a degree) defined by the noise level of the tracker. Thus, fixations were defined jointly by position and velocity. Blinks were detected by occlusion of the pupil and the cursor was occluded during the blink.

### Procedure

Participants wore an eye tracker mounted on the head, and were seated in front of a computer screen.

Participants started with a “free viewing” task in which they were asked to look at a scene on a computer screen for 10 s. Then, participants performed an “active viewing” task: picturing themselves making a butter and jelly sandwich and pouring a glass of water in front of a computer screen. In the imagined movement task (active viewing), timing began when the experimenter said “go” (when the scene was displayed), and stopped when the participant said “stop” upon completing imagined action.

Before the experiments, the layout was occluded by a black screen showing the five calibration points, enabling the participants to be calibrated on the plane of the working surface. The participant had to fixate the targets (white dots) while his/her eye positions were recorded by the system. Using these reference points, the system creates a mapping function that relates all eye positions to points in the calibration area. Once the calibration was completed, this was removed, and participant immediately started the task. A re-calibration procedure was performed after each task. The entire session lasted approximately 30 min; the calibration time depending on participants.

### Data analysis

Temporal and spatial characteristics of gaze patterns were extracted for each group of participants and each task. We measured the total gaze duration on specific objects, in instances where several successive fixations were made on the same object. Gaze duration on both relevant and irrelevant objects was determined. Eye movement variables were submitted to analyses of variance (ANOVA) using the STATISTICA software from StatSoft (version 7.1, F-94700 Maisons-Alfort, France). Possible confounding effects of medication, illness duration, positive and negative symptom categories (indexed by the PANSS), patient categories (in- and out-patients), gender were examined. For patients with schizophrenia, there were no significant correlations between medication, illness duration or symptom category on one hand and any of the scan path variables on the other. Relationships between gender and scan path variables were computed for both groups but no statistically significant correlations emerged.

## Results

### Gaze durations on relevant objects (RO) and irrelevant objects (IO)

**Figure 2:**
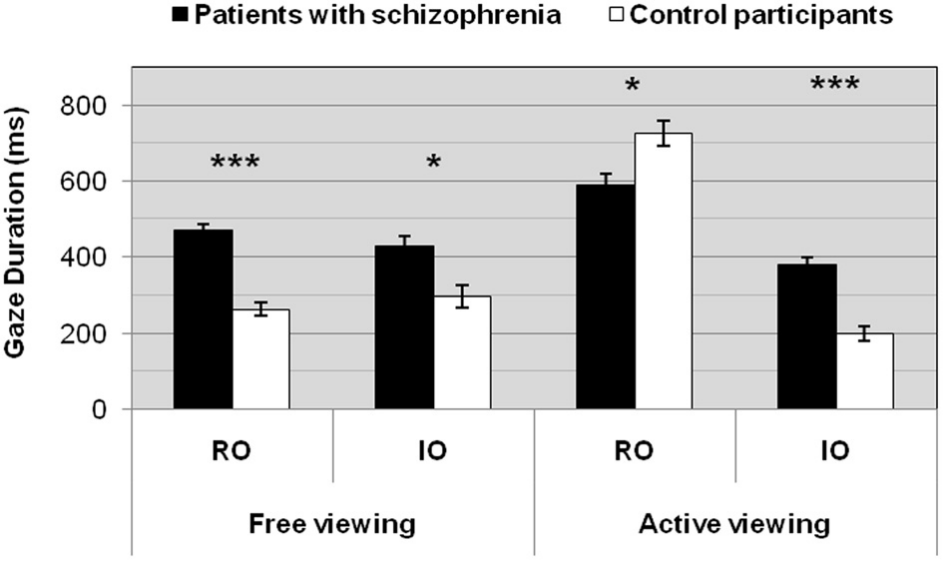
**Gaze durations for patients and controls as a function of the task**. ^*^p < 0.01; ^***^p < 0.0001. RO, relevant objects; IO, irrelevant objects.

A 2 (group: Patients/Controls) × 2 (task: Free/Active viewing) × 2 (objects: Relevant/Irrelevant) repeated measures multivariate ANOVA showed a significant main effect of group [*F*_(1, 34)_ = 37.0, *p* < 0.0001], task [*F*_(1, 34)_ = 38.9, *p* < 0.0001], and objects [*F*_(1, 34)_ = 79.0, *p* < 0.0001] on the gaze durations. There was also a significant interaction between group, task and objects [*F*_(1, 34)_ = 34.6, *p* < 0.0001] (Figure 2).

#### Free viewing condition

Patients with schizophrenia exhibited longer gaze durations than controls on objects in the free viewing condition [RO: *F*_(1, 34)_ = 63.3, *p* < 0.0001 and IO: *F*_(1, 34)_ = 10.9, *p* < 0.003].

Patients and controls looked equally at relevant and irrelevant objects in the free viewing condition [Patients, RO vs. IO, *F*_(1, 34)_ = 1.4, *p* = 0.2, ns – Controls, RO vs. IO, *F*_(1, 34)_ = 0.9, *p* = 0.4, ns].

#### Active viewing condition

Patients with schizophrenia exhibited longer gaze durations than controls on irrelevant objects in the active viewing condition [IO: *F*_(1, 34)_ = 40.5, *p* < 0.0001], whereas controls made longer gaze durations on relevant objects in the same condition [RO: *F*_(1, 34)_ = 9.6, *p* < 0.004].

Patients and controls looked more at relevant than at irrelevant objects in the active viewing condition [Patients, RO vs. IO, *F*_(1, 34)_ = 27.3, *p* < 0.0001 – Controls, RO vs. IO, *F*_(1, 34)_ = 174.5, *p* < 0.0001].

#### Free vs. active viewing conditions

As controls, patients looked more at relevant objects in the active viewing condition [Patients, RO: free vs. active viewing, *F*_(1, 34)_ = 12.8, *p* < 0.002 – Controls, RO: free vs. active viewing, *F*_(1, 34)_ = 196.1, *p* < 0.0001], but unlike controls, patients with schizophrenia did not show any significant difference on irrelevant objects between the free viewing and the active viewing condition [IO: free vs. active viewing, *F*_(1, 34)_ = 1.9, *p* = 0.2, ns], whilst controls made fewer gaze durations on irrelevant objects in the active viewing condition [IO: free vs. active viewing, *F*_(1, 34)_ = 7.5, *p* < 0.01].

**Figure 3:**
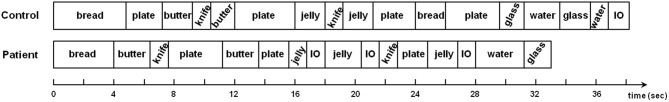
**Sequence of objects fixated to accomplish the task (active viewing) for one participant of each group**. IO, irrelevant object.

An example of one participant's fixations for each group is given in Figure 3. Each participant began to make a series of fixations on the task-relevant objects as the bread, the butter, the knife or the plate. The order in which the relevant objects to accomplish the task were fixated was the same for patients and controls.

### Performance task duration

A 2 (group: Patients/Controls) × 1 (task: Active viewing) ANOVA showed no significant difference between the two groups on the total task duration [*F*_(1, 34)_ = 0.1, *p* = 0.8]. For accomplishing the imagined task, patients needed on average 27 s and controls needed 28 s.

## Discussion

Eye movements of patients with schizophrenia and healthy control participants were recorded both under a free viewing condition and under an active viewing condition with scene image as stimuli. In the active viewing condition, participants were instructed to imagine making accurate movements between different task-relevant objects.

Consistent with previous eye movement studies using images as stimuli (e.g., Bestelmeyer et al., 2006), abnormalities in patients with schizophrenia were found in the free viewing condition. Patients showed longer fixation durations, and fewer eye fixations, than healthy control participants in the free viewing condition, but they did not differ from control participants in the active viewing condition for temporal scan path variables. This result can be related to studies on attentional control and cognitive flexibility in schizophrenia (Granholm et al., 1999; Ducato et al., 2008a,b). Indeed, patients with schizophrenia are able to normalize their pattern of visual exploration when they are actively involved in more demanding tasks (Kurachi et al., 1994; Tonoya et al., 2002; Delerue et al., 2010; Delerue and Boucart, 2012, 2013).

Our results show that the patients' visual pattern improved in the active viewing condition. However, for spatial scan path variables, patients looked more at task-irrelevant objects (distractors) than controls in the active viewing condition in which participants were asked to picture themselves making a sandwich in moving eyes to task-relevant objects on a scene image. This difference in the spatial distribution of fixations between the two groups in the imagined task raises the possibility that patients with schizophrenia did not perform the imagined motor task correctly. However, an aspect of the results suggests otherwise. Indeed, the order in which the relevant objects to accomplish the task were fixated was the same for patients and controls. In addition to recording eye movements, the time to execute the imagined task was measured as a means of ensuring that the task was performed in an equivalent manner between the two groups of participants. The results showed no significant difference between the two groups on the imagined task duration. This suggests that patients were complying with instructions in the imagined task.

Visual search has been found to be modulated by action intentions in healthy participants (Bekkering and Neggers, 2002; Hannus et al., 2005). Abnormalities in action have been observed in various forms in patients with schizophrenia. Clinically, they appear as poverty of action, disorganized behavior, stereotyped, and incoherent actions. Behavioral experimental studies have reported disturbances of action production, whether an action has to be explicitly performed, like figure copying tasks (Jogems-Kosterman et al., 2001; Grootens et al., 2009) or planning of motor sequences (Delevoye-Turrell et al., 2007) or not, like intention of action, observation of action, or manipulation of action knowledge. Zalla et al. (2001, 2006) investigated the ability of patients with schizophrenia to organize action knowledge and elaborate a plan of action. The authors found that patients with schizophrenia performed significantly worse than controls, and frontal lobe patients, in an action verbal generation task. They suggested that patients with schizophrenia are impaired in the sequential organization of action events.

Other studies (Danckert et al., 2002b; Maruff et al., 2003) have assessed the ability of patients with schizophrenia to perform a motor imagery task. These studies have reported deficits in motor imagery in these patients. The degree of impairment in imagined movements was not correlated with symptom profile (Danckert et al., 2002b). However, in a separate study, patients with and without passivity delusions were tested using a similar motor imagery task, and only the patients with passivity delusions showed a specific impairment in the execution of imagined motor sequences (Maruff et al., 2003). These authors have found a deficit in the ability to generate or make use of internal model of intended actions. This impairment is likely to reflect dysfunction in parietal association cortices that have been shown to be crucial for making use of internal models of goal-directed movements (Sirigu et al., 1995; Danckert et al., 2002a).

Compared with healthy participants, the visual exploration pattern of patients could be explained by their susceptibility to distraction. Indeed, it is known that patients with schizophrenia exhibit a higher sensitivity to distraction that healthy observers (Ducato et al., 2008a,b). In our study, it might be that a salient feature in the object (e.g., color, shape, or contrast edges) automatically captured the patients' attention and that they found it difficult to disengage their attention from that salient feature. Impaired spatial attention, appearing in longer duration of attentional disengagement, has been reported in patients with schizophrenia with the Posner's paradigm (Posner et al., 1988; Wigal et al., 1997). Moreover, studies using the antisaccade paradigm have demonstrated that patients show difficulty inhibiting a reflexive saccade (Hutton and Ettinger, 2006).

The patients' visual exploration may also be viewed in the framework of Gestalt dysfunctions in schizophrenia. Indeed, a majority of studies showed impairments of perceptual organization in schizophrenia (Uhlhaas and Silverstein, 2003, 2005; Silverstein and Uhlhaas, 2004; Brand et al., 2005), indicating that many patients with schizophrenia appear to have deficient gestalt perception.

One possible limitation of this study is that only one trial per condition was used. Thus, measures of reliability come from average performance over fixations within a task, and similarity between subjects, as indicated by the standard error measures. Moreover, only a small sample of participants (2 × 18) has been tested. The magnitude of the reported effects indicate that this is not a major concern, however. This is an exploratory pilot study, and future studies on different imagining tasks are needed to confirm the presented results. Another limitation is that although in our study, antipsychotic dosage equivalents did not appear to be correlated to various eye movement variables, we cannot exclude an affect of medication. Kojima et al. (1990) have reported that neither the number of fixations nor scan path length were correlated with the chlorpromazine-equivalent dosage in 50 chronic schizophrenic patients, and this was confirmed by Matsushima et al. (1992). Streit et al. (1997) and Loughland et al. (2002a,b) found no relation between dysfunctional scan paths in schizophrenia and medication, whilst Williams et al. (2003) reported that patients treated with risperidone showed greater attention to salient features. Reilly et al. (2008) showed that pharmacological treatment might have an effect on eye movement control in saccadic tasks, and Giersch et al. (2010) reported effects of benzodiazepines on cognitive functions.

The present study shows that patients with schizophrenia improve their visual exploration under task-driven attentional conditions, in an imagined task that is an example of embodied cognition. However, our results also show that patients with schizophrenia have some difficulties to generate accurate internal representation of goal-directed actions.

### Conflict of interest statement

The authors declare that the research was conducted in the absence of any commercial or financial relationships that could be construed as a potential conflict of interest.
